# A high-density genetic map construction and sex-related loci identification in Chinese Giant salamander

**DOI:** 10.1186/s12864-021-07550-0

**Published:** 2021-04-01

**Authors:** Qiaomu Hu, Yang Liu, Xiaolin Liao, Haifeng Tian, Xiangshan Ji, Jiajie Zhu, Hanbing Xiao

**Affiliations:** 1 Yangtze River Fisheries Research Institute, Chinese Academy of Fishery Sciences, Wuhan, 430223 Hubei China; 2 Laboratory for Marine Fisheries Science and Food Production Processes, Pilot National Laboratory for Marine Science and Technology (Qingdao), Yellow Sea Fisheries Research Institute, Chinese Academy of Fishery Sciences, Qingdao, China; 3 Key Laboratory of Ecological Impacts of Hydraulic-Projects and Restoration of Aquatic Ecosystem of Ministry of Water Resources, Institute of Hydroecology, Ministry of Water Resources and Chinese Academy of Sciences, Wuhan, 430079 China; 4 Shandong Provincial Key Laboratory of Animal Biotechnology and Disease Control and Prevention, Shandong Agricultural University, Taian, 271018 Shandong China; 5 Guangxi Academy of Fishery Sciences, Nanning, 530021 Guangxi Province China

**Keywords:** Chinese giant salamander, ddRAD, SNP, High-density linkage map, Sex-related loci mapping

## Abstract

**Background:**

The Chinese giant salamander *Andrias davidianus* is an important amphibian species in China because of its increasing economic value, protection status and special evolutionary position from aquatic to terrestrial animal. Its large genome presents challenges to genetic research. Genetic linkage mapping is an important tool for genome assembly and determination of phenotype-related loci.

**Results:**

In this study, we constructed a high-density genetic linkage map using ddRAD sequencing technology to obtain SNP genotyping data of members from an full-sib family which sex had been determined. A total of 10,896 markers were grouped and oriented into 30 linkage groups, representing 30 chromosomes of *A. davidianus*. The genetic length of LGs ranged from 17.61 cM (LG30) to 280.81 cM (LG1), with a mean inter-locus distance ranging from 0.11(LG3) to 0.48 cM (LG26). The total genetic map length was 2643.10 cM with an average inter-locus distance of 0.24 cM. Three sex-related loci and four sex-related markers were found on LG6 and LG23, respectively.

**Conclusion:**

We constructed the first High-density genetic linkage map and identified three sex-related loci in the Chinese giant salamander. Current results are expected to be a useful tool for future genomic studies aiming at the marker-assisted breeding of the species.

**Supplementary Information:**

The online version contains supplementary material available at 10.1186/s12864-021-07550-0.

## Background

The Chinese giant salamander *Andrias davidianus*, historically widely distributed in China, is the world’s largest extant amphibian. Due to its habitat destruction and hunting by man, it has been classified as endangered by the International Union for Conservation of Nature and Nature Resources since the 1980s [1]. To conserve the wild resources of giant salamander, artificial breeding technology was developed and succeeded [2, 3]. Because of its palatability and nutritional value, the Chinese salamander is commercially cultured and amphibians are now allowed to reach the market [4]. It is considered an important species to study because scientists see it as a living fossil present on Earth since 350 million years ago and linking aquatic to terrestrial organisms, with complex sex determination and differentiation characteristics. Until now, most studies on *A. davidianus* focused on the immune mechanisms [5–8], and only few papers were reported for the sex-differentiation issues [9–11].

Previous studies reported that the Chinese giant salamander has a huge genome (about 50 Gb) which was a big challenge to assemble [12], and to this direction the construction of a genetic linkage map will be a valuable tool. In the previous study, Simple sequence repeats (SSR) was the mainly basic tools to generate a linkage map in various species. With the development of biotechnology, single nucleotide polymorphisms (SNP) has become the most popular tools to construct the high-density linkage maps, because of their widely distribution in the genome and development of next genomic sequence technology. Recently, high-density genetic linkage maps have become increasingly important and widespread in cultured aquatic species. In fish, genetic linkage maps have been reported in *Salmo salar* [13, 14], *Cyprinus carpio* [15], *Paralichthys olivaceus* [16], *Megalobrama amblycephala* [17], *Larimichthys polyactis* [18, 19], *Oreochromis niloticus* [20], *Ictalurus punctatus* [21], and *Lates calcarifer* [22], all of which possess relatively small genomes. In amphibian, genetic linkage maps have been constructed in several species including *Rana temporaria* [23], *Hyla arborea* [24], and *Lissotriton* newts [25]. However, high-density genetics maps are not yet available in amphibians.

Quantitative trait locus (QTL) mapping is an effective means to relate phenotype to the genome. In previous study, QTL mapping has been used in various species for different traits location including loci related to ecomic phenotype [26] and resistance to disease [27, 28]. The composite interval mapping (CIM) is a common method for mapping QTLs based on linkage map [29] and it was used to located binary trait such as sex [30] and metamorphosis-related traits loci in fish [31]. To identify the sex chromosome of *A. davidianus* without a genome, the CIM method was effective approach. In the previous study, we explored the female-specific marker to identify the genetic sex of the salamander which was important to detect the sex reversal salamander treated by high temperature and sex hormone in the study of sex determination mechanism [32, 33]. we also characterized sex related gene in the sex differentiation [34, 35]. Studies on the genetic level are necessary to identify sex related regions and ascertain the sex determination mechanism. In the present study, we used a single cultured *A. davidianus* full-sib family to construct a high-density linkage map based on the ddRAD sequencing. Using the constructed linkage map. We identified three sex-related loci in different linkage groups. These results provide an effective genetic tool for *A. davidianus* genome assembly and marker-assisted breeding.

## Results

### Library construction and sequencing

Ninety-seven RAD-seq libraries from two parents and 95 offspring were constructed and sequenced on an Illumina HiSeq X platform to generate raw reads. Based on the data trimming, 10.49 billion reads comprising ∼1516.07 Gb of sequencing data were individually partitioned into RAD tags according to their multiplex identifiers (GenBank accession No. SRP155453). Finally, female and male parent datasets which contained 210,000,000 and 190,000,000 clean reads, respectively, were correspondingly partitioned into 1,887,649 and 6,378,461 RAD tags. A total of 1458.71 Gb of data (mean 15.35 Gb per individual, 51 M reads pair per sample) were produced and divided into 302,909,245 RAD tags (ranging from 1,934,524 to 6,800,866 with a mean of 3,188,518.37) from the 95 offspring, for individual SNP discovery (Table S1).

### SNP discovery and genotyping

RAD tags from each individual were clustered and compared, after stringent selection by the above-described method. A custom k-mer matching algorithm that excluded exact sequence matches (monomorphic loci) per 41 bp sequence was used to identify the SNP of the parents contained the RAD tags. A panel of 10,896 high-fidelity SNPs in both parents was identified, and alleles for each marker were assigned to their respective parent donor. The SNPs were analyzed across the 95 offspring with genotyping data and classified into three categories using an in-house script: maternal heterozygous (3343 SNPs), paternal heterozygous (6066 SNPs), and heterozygous in both (1487 SNPs) (Table S2).

### High-density linkage map construction

**Fig. 1:**
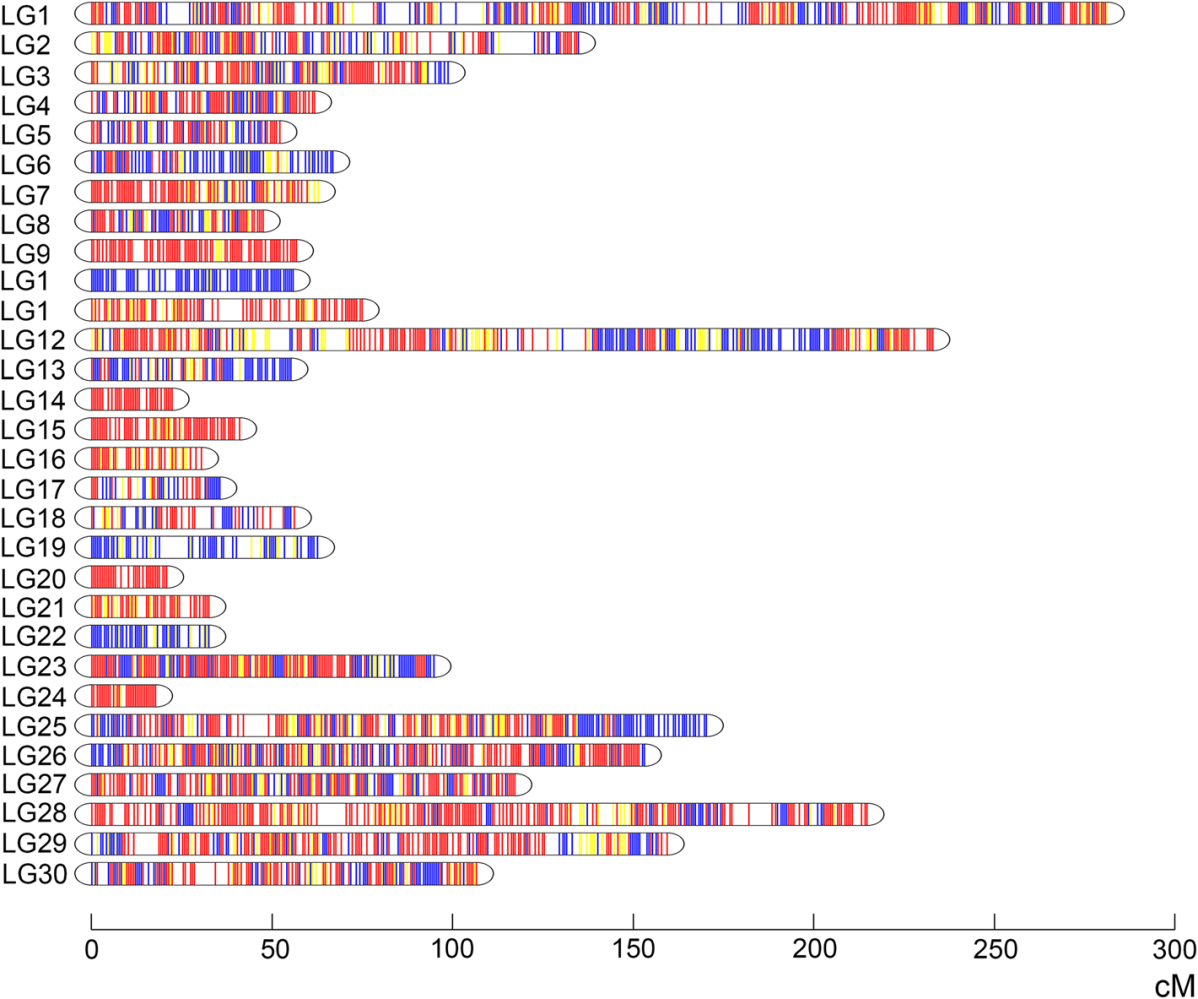
Integrated genetic linkage maps of *Andreas davidianus* comprising 10,896 markers assigned to 30 linkage groups (LG1–LG30). Genetic distances in Kosambi centiMorgans are listed on the left side of the linkage groups, and markers are listed on the right side of the linkage groups

In the present study, Join-map and Lep-Map were used to construct the high-density genetics linkage map of *A. davidianus*. In both the maternal and paternal map, a total of 10,896 segregating SNPs were successfully classified into 30 linkage groups which was consistent with the karyotype 2n = 60 (Fig. 1). The maternal map contained 4830 SNPs with a total genetic distance of 2580.04 cM., The length of LGs ranged from 0 cM (LG27) to 298.26 cM (LG1) with an average genetic length of 86.00 cM (Table 1). Seven thousand five hundred fifty-three SNPs representing a total length of 2011.80 cM ranging from 0 cM (LG21, LG27, and LG30) to 244.33 cM (LG1) with an average genetic length of 67.06 cM were consisted the corresponding paternal map. The longest linkage group was LG1 which was 244.33 cM and 298.26 cM in maternal and paternal map, respectively. However, the shortest was 0 cM distributed in LG21, LG27, and LG30 in the paternal map and LG27 in the maternal map.

**Table 1:** The characterization of genetic linkage groups in Chinese giant salamander

LG ID	Paternal map			Maternal map			Integrated map		
	No. of SNPs	Distance (cM)	Average inter-loci distance (cM)	No. of SNPs	Distance (cM)	Average inter-loci distance (cM)	No. of SNPs	Distance (cM)	Average inter-loci distance (cM)
1	674	244.33	0.36	476	298.26	0.63	893	232.59	0.26
2	598	190.49	0.32	468	250.72	0.54	990	280.81	0.28
3	571	126.48	0.22	399	91.29	0.23	878	94.61	0.11
4	479	207.83	0.43	461	142.50	0.31	819	169.94	0.21
5	482	95.61	0.20	324	206.01	0.64	715	152.80	0.21
6	490	107.73	0.22	294	146.24	0.50	684	116.99	0.17
7	559	136.63	0.24	197	257.03	1.30	681	214.38	0.31
8	405	108.35	0.27	196	213.29	1.09	541	159.12	0.29
9	379	91.67	0.24	225	113.66	0.51	522	106.37	0.20
10	333	85.95	0.26	231	135.56	0.59	484	134.56	0.28
11	341	74.49	0.22	195	115.06	0.59	458	98.52	0.22
12	218	50.91	0.23	133	56.84	0.43	314	61.57	0.20
13	161	46.02	0.29	98	47.73	0.49	240	52.01	0.22
14	87	66.21	0.76	169	17.44	0.10	228	66.64	0.29
15	215	26.67	0.12	42	54.99	1.31	225	62.60	0.28
16	134	36.53	0.27	89	41.67	0.47	202	47.38	0.23
17	192	0.00	0.00	8	25.45	3.18	192	56.57	0.29
18	20	34.47	1.72	183	28.12	0.15	183	55.65	0.30
19	178	32.53	0.18	27	62.40	2.31	180	74.74	0.42
20	59	43.77	0.74	138	38.81	0.28	173	55.06	0.32
21	156	0.00	0.00	2	14.25	7.13	156	22.19	0.14
22	152	1.01	0.01	10	15.17	1.52	152	40.87	0.27
23	148	24.67	0.17	42	28.41	0.68	148	30.32	0.20
24	58	14.21	0.25	89	45.28	0.51	136	35.40	0.26
25	87	36.90	0.42	73	45.41	0.62	134	56.00	0.42
26	28	70.95	2.53	129	36.38	0.28	129	62.41	0.48
27	117	0.00	0.00	0	0.00	NA	117	20.68	0.18
28	111	19.69	0.18	23	36.57	1.59	111	32.36	0.29
29	16	37.70	2.36	106	12.47	0.12	106	32.35	0.31
30	105	0.00	0.00	3	3.03	1.01	105	17.61	0.17
Total	7553	2011.80	0.27	4830	2580.04	0.53	10,896	2643.10	0.24

Finally, the integrated map consisting of 30 LGs was constructed by 10,896 effective markers. The total length of the integrated map was 2643.10 cM. The average number of effective loci was 363 distributed in genetic distance of 88.10 cM with an average inter-locus distance of 0.24 cM. The genetic distance of LGs ranged from 17.61 cM (LG30) to 280.81 cM (LG1), and a mean inter-locus distance ranged from 0.11 (LG3) to 0.48 cM (LG26). The maximum density of SNP marker in linkage group was LG3 containing 878 effective loci and minimum density was LG30 containing 105 effective loci (Table S3).

### Sex-related loci

**Fig. 2:**
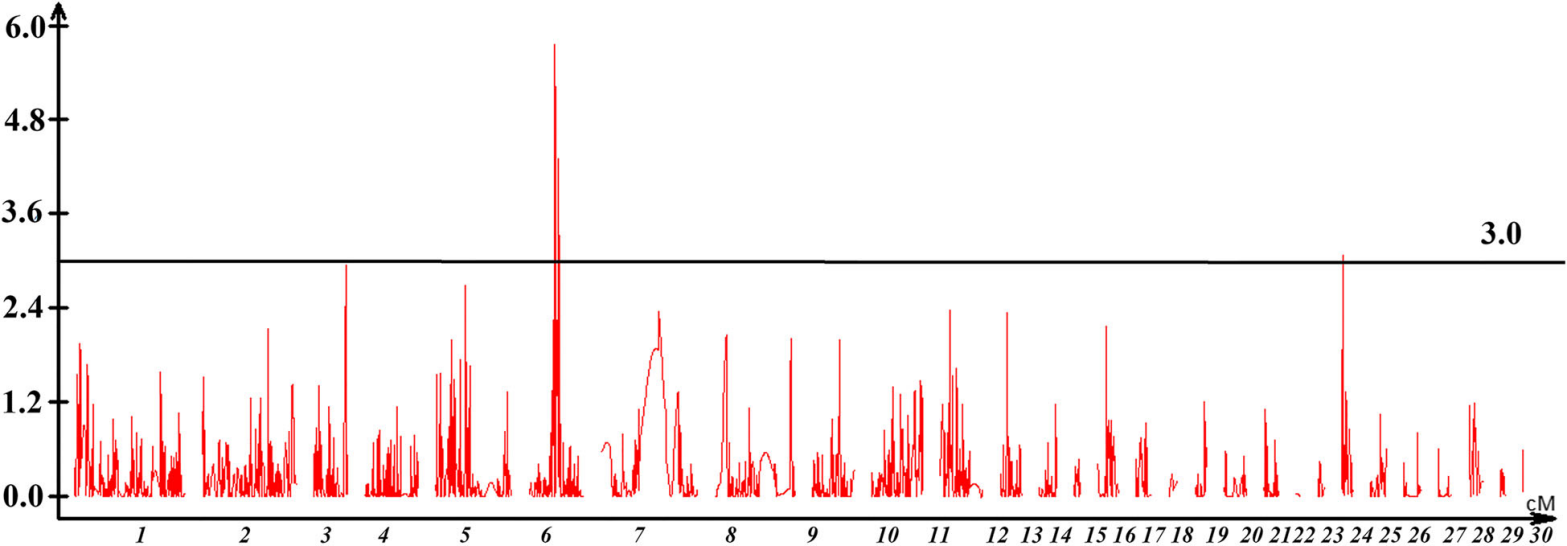
Sex-related loci of *Andreas davidianus*. The black horizontal line represents a linkage group-wise logarithm of odds significance threshold of 3.0

Sex-related loci of *A. davidianus* were detected using CIM on the maternal and paternal map by WinQTL cart 2.5. Total of 3 loci were found on the maternal map LG6 (qs-1, qs-2) and LG11(qs-3). The likelihood of odd (LOD) of the loci were 5.8, 4.3, and 3.1, which explained 29.37, 12.00, and 6.78% of the phenotypic variation, respectively. Genotyping results of SNP 97.nn_np_297384, 99.nn_np_294732, 99.nn_np_278109, and 90.hk_hk_96246 in the sex-related loci were correlated with phenotype by the single-marker analysis of the WinQTL cart 2.5 (Table 2, Fig. 2). Eight maternal SNPs were found in the sex-related loci qs-1 and qs-2. The correlation of the eight SNPs with phenotypic sex ranged from 57.81% (99.nn_np_278 109) to 68.18% (99.nn_np_282 501) (Table 3).

**Table 2:** Parameter value of loci and estimation of genetic effects

Locus	Linkage group	Covered area cM	Associated marker	Peak of LOD	Variation %
qS-1	LG6	64.9–68.5	97.nn_np_297384,99.nn_np_294732	5.8	29.37
qS-2	LG6	76.2–77	99.nn_np_278109	4.3	12
qS-3	LG23	2.0–2.1	90.hk_hk_96246	3.1	6.78

**Table 3:** Correlation of markers in sex-related loci with phenotype

Marker ID	Linkage group	Position	Match with Phenotype	Mismatch with Phenotype	Missing	Correlation
99.nn_np_263559	LG6	64.94	68	25	2	63.24%
99.nn_np_139095	LG6	64.94	61	24	10	60.66%
99.nn_np_282501	LG6	65.95	66	21	8	68.18%
99.nn_np_179118	LG6	65.95	60	22	13	63.33%
97.nn_np_297384	LG6	66.96	63	22	10	65.08%
99.nn_np_294732	LG6	66.96	69	24	2	65.22%
99.nn_np_209757	LG6	76.12	60	24	11	60.00%
99.nn_np_278109	LG6	76.12	64	27	4	57.81%

## Discussion

Genetic linkage mapping is an important tool in genomic and genetic studies [36, 37]. Abundant molecular markers with an established order of arrangement provide an excellent resource for breeding research. Most amphibians possessed a large genome [38, 39], which exhibited a challenge in constructing a high-density genetic linkage map. Research exploring SNPs in *A. davidianus* has been reported, but identified markers were not used to build a linkage map [40]. Sessions et al. assembled thirty linkage groups in maternal, paternal, and integrated maps, showing consistency with the karyotype [41]. *Andrias davidianus* possesses a genome of ~ 50 G [12], hampering genomic and genetic study because of lack of large genome assembly technology and linkage map. The present study exhibited the first high-density genetic linkage map using SNPs of *A. davidianus*.

Thirty linkage groups were constructed with the average marker-interval of 0.24 cM, which was the highest density linkage map in amphibians. In common frog, 107 SSR markers was distributed in 15 linkage groups with total length 1698.8 cM [23]. In Lissotriton newts, total of 1146 markers was distributed in 12 linkage groups with an average marker interval of 1.29 cM [25]. The highest density linkage map of fish species was constructed for channel catfish, with the average marker interval of 0.22 cM [21]. Hence, the *A. davidianus* SNP linkage map displays high density even compared to fish species with relatively small genomes. In this study, we constructed 0 cM linkage groups in maternal and paternal map respectively. The main reason may be the specificity of *A. davidianus* genome. Some types of markers with different genotype distributed on different location in one chromosome. However, the other types of markers obtained the same genotype from same individual distributing on same location in linkage group (0 cM). These results indicated the LD differ in maternal and paternal linkage groups.

The high-density linkage map provided the means of fine mapping *A. davidianus* sex-related QTL. Three loci were identified on the maternal map LG6 (qs-1, qs-2) and LG11 (qs-3). The genetic distance of the QTL locus ranged from 0.1 cM to 3.5 cM suggested a narrow regions in the linkage group. This may provide valuable information for sex-related gene detection. While the same method was used to identify the loci on the maternal and paternal maps, all observed sex-related loci were on the maternal map. It is possible that some key sex-related markers were homozygous on the paternal map. Sex chromosomes have a greater number of repeat sequences, making the assembly of sex chromosomes and the construction of a sex linkage group more difficult, and sex is readily influenced by factors other than chromosome [42]. Some sex-related QTLs have been detected in other species, seven in the half-smooth tongue sole with five sex-linked markers [43], and 175 sex-related regions in turbot, most distributed among three LGs, most likely to be the sex chromosome [44]. In the present study, the major loci were detected only on linkage group LG6, providing evidence for the sex chromosome of *A. davidianus*. We analyzed sex related markers in this region and found correlation coefficient which ranged from 57.81 to 68.18% between phenotype and genotype in eight maternal SNP marker. Possibly due to low sequencing depth, some key SNPs in sex-related loci were not mapped on the linkage group. This region was considered as sex-related loci. So many SNPs in this region was related with sex too [17]. Verification could be carried out to confirm sex-linked genes and markers in *A. davidianus* through expanded single-linkage data in future. Further research requires expanded knowledge of the *A. davidianus* genome.

## Conclusions

We constructed a high-density genetic linkage map using ddRAD sequencing technology to obtain SNP genotyping data from one full-sib family. A total of 10,896 markers were grouped and oriented into 30 linkage groups (LG), representing 30 chromosomes of *A.davidianus*. The genetic length of LGs ranged from 17.61 cM (LG30) to 280.81 cM (LG1), with a mean inter-locus distance ranging from 0.11(LG3) to 0.48 cM (LG26). The total genetic map length was 2643.10 cM with an average inter-locus distance of 0.24 cM. At last, we found three sex related loci on the maternal map LG6 (qs-1, qs-2) and LG11(qs-3). Genotyping results of four SNP markers (97.nn_np_297384, 99.nn_np_294732, 99.nn_np_278109, and 90.hk_hk_96246) in the sex-related loci were significantly correlated with phenotype which indicated the four markers were sex-related markers. We constructed a High-density genetic linkage map and mapped 3 sex-related loci, which provides the linkage map is a useful tool for genomic study and provides a genetic basis for marker-assisted breeding of Chinese giant salamander.

## Methods

### Mapping population

Total of 97 apparently healthy *A. davidianus* including two parents (8 year old) and 95 salamander larvae (body weight 34.24 ± 12.13 g; body length 17.85 ± 1. 76 cm) from one F3 full-sib family were collected from Zhejiang Yongqiang Chinese Giant Salamander Limited Company (Jinhua, Zhejiang province, China). The salamanders were soaked in 4 L MS222 with the concentration of 0.5 g/L for 10mins to anaesthetize. After all the salamanders were unconscious, the vertebra was broken from the neck, then the skin of the neck was removed, and muscle tissue was collected. All operations were carried out as per Yangyze River Fisheries Research Institute Care Committee (no. 2013001). The muscle tissue was collected from each salamander using the tissue DNA extracting kit (Omega, USA) to extract genomic DNA following manufacturer’s instruction. The concentration and quality of DNA were detected by Agilent 2100 Bioanalyzer (Agilent Technologies, Santa Clara, CA) and 1% agarose gel electrophoresis. Genetic sex of each offspring was identified using a sex-specific marker explored by our lab [32].

### RAD library construction, sequencing, and genotyping

The RAD library construction, sample indexing, and pooling followed the natural population [43, 45]. The high quality genomic DNA (250 ng) from each of the 97 individuals were used to construct libraries. The genomic DNA were digested by enzyme EcoRI and NIaIII (New England Biolabs) at 37 °C for 1 h and then at 65 °C for 30 min to inactivate the enzyme. Illumina HiSeq X on a total throughput of 24 lanes (~60Gb ddRAD data for each lane) were used to perform the Pair-end (150 bp) sequencing. Raw sequence reads were trimmed to 110 nucleotides from the 3′ end to ensured a quality value >Q30 in more than 98% of the nucleotides (equals 0.1% sequencing error). To produce unique candidate alleles for each RAD locus, Stacks [46] had been used to cluster the trimmed reads by sequence similarity. A maximum base-pair mismatch of one was allowed in this step for the genetic mapping population. Stacks were used under default parameters to collapse the RAD-tags into clusters for SNP calling. High-Quality and Bi-allelic SNPs were identified under following criteria which were 1) > =6 and < =100 reads for each loci, otherwise this loci will be considered as missing data, 2) present in at least 60% (< 39 genotyping-deletion rate) of the individuals.

### Linkage map construction

RAD-based SNPs were first tested against the expected segregation ratio at the beginning of the linkage analysis. Markers were filtered out by a chi-square test for separation ratio at the intersection sites with *P* < 0.001. The linkage map was constructed using joinmap and Lep-Map. Joinmap was used to divide markers into groups using a grouping tree function in CP model with LOD = 8.0 [47] and Lep-MAP was used to determine marker order [48]. Kosambi mapping function was used to estimate of each linkage group distances.

### Sex-related loci mapping

CIM method of WinQTL Cart 2.5 software was used to map the sex-related loci on the male and female linkage maps respectively [49]. Model six with three parameters as 10-cM window size, five control markers, and a step size of 1-cM was used to analyze the forward and backward stepwise regression. The location of each QTL was determined based on its LOD peak location and surrounding region with the threshold value of 3.0. Single-marker association tests with WinQTL Cart 2.5 was used to analysis the association between phenotype and genotype for each marker [49]. All maternal SNPs in the located sex-related region were analyzed to detect correlation with the phenotype to find sex-related SNPs.

## Supplementary Information

**Additional file 1: Table S1**. Original data of each sample for RAD sequencing

**Additional file 2: Table S2**. Genotyping information and location of each SNP on the 30 LGs

**Additional file 3: Table S3** Distribution of each SNP on the 30 LGs

## Data Availability

All data generated or analyzed during this study are included in this article and its supplementary information files. GenBank accession No. SRP155453.

## Abbreviations

LG: Linkage group
QTL: Quantitative trait locus
ddRAD: Double digest restriction-site associated DNA
SNP: Single-nucleotide polymorphism
LOD: Likelihood of odd
CIM: Composite interval mapping
